# Wild Animals Are Reservoirs and Sentinels of *Staphylococcus aureus* and MRSA Clones: A Problem with “One Health” Concern

**DOI:** 10.3390/antibiotics10121556

**Published:** 2021-12-20

**Authors:** Idris Nasir Abdullahi, Rosa Fernández-Fernández, Guillermo Juárez-Fernández, Sandra Martínez-Álvarez, Paula Eguizábal, Myriam Zarazaga, Carmen Lozano, Carmen Torres

**Affiliations:** Area of Biochemistry and Molecular Biology, One-Health Research Group, University of La Rioja, 26006 Logroño, Spain; idris-nasir.abdullahi@unirioja.es (I.N.A.); rosa.fernandez.1995@gmail.com (R.F.-F.); gukle.juarez@gmail.com (G.J.-F.); sandra.martinezal@unirioja.es (S.M.-Á.); paulaeguimar@gmail.com (P.E.); myriam.zarazaga@unirioja.es (M.Z.); carmen.lozano@unirioja.es (C.L.)

**Keywords:** wild animals, MRSA-CC398, *mecC*-MRSA, livestock-associated MRSA, nasal carriage, bacterial zoonosis

## Abstract

*Background:* The availability of comprehensive data on the ecology and molecular epidemiology of *Staphylococcus aureus*/MRSA in wild animals is necessary to understand their relevance in the “One Health” domain. *Objective:* In this study, we determined the pooled prevalence of nasal, tracheal and/or oral (NTO) *Staphylococcus aureus* (*S. aureus*) and methicillin-resistant *S. aureus* (MRSA) carriage in wild animals, with a special focus on *mecA* and *mecC* genes as well as the frequency of MRSA and methicillin susceptible *S. aureus* (MSSA) of the lineages CC398 and CC130 in wild animals. *Methodology:* This systematic review was executed on cross-sectional studies that reported *S. aureus* and MRSA in the NTO cavities of wild animals distributed in four groups: non-human primates (NHP), wild mammals (WM, excluding rodents and NHP), wild birds (WB) and wild rodents (WR). Appropriate and eligible articles published (in English) between 1 January 2011 to 30 August 2021 were searched for from PubMed, Scopus, Google Scholar, SciElo and Web of Science. *Results:* Of the 33 eligible and analysed studies, the pooled prevalence of NTO *S. aureus* and MRSA carriage was 18.5% (range: 0–100%) and 2.1% (range: 0.0–63.9%), respectively. The pooled prevalence of *S. aureus*/MRSA in WM, NHP, WB and WR groups was 15.8/1.6, 32.9/2.0, 10.3/3.4 and 24.2/3.4%, respectively. The prevalence of *mecC*-MRSA among WM/NHP/WB/WR was 1.64/0.0/2.1/0.59%, respectively, representing 89.9/0.0/59.1/25.0% of total MRSA detected in these groups of animals.The MRSA-CC398 and MRSA-CC130 lineages were most prevalent in wild birds (0.64 and 2.07%, respectively); none of these lineages were reported in NHP studies. The MRSA-CC398 (mainly of *spa*-type t011, 53%), MRSA-CC130 (mainly of *spa* types t843 and t1535, 73%), MSSA-CC398 (*spa*-types t571, t1451, t6606 and t034) and MSSA-CC130 (*spa* types t843, t1535, t3625 and t3256) lineages were mostly reported. *Conclusion:* Although the global prevalence of MRSA is low in wild animals, *mecC*-mediated resistance was particularly prevalent among MRSA isolates, especially among WM and WB. Considering the genetic diversity of MRSA in wild animals, they need to be monitored for effective control of the spread of antimicrobial resistance.

## 1. Introduction

Antimicrobial resistance (AMR) constitutes one of the major global health challenges of the twenty-first century. The holistic approach, “One Health”, is being considered as an important tool to avoid the emergence and spread of multi-drug resistant bacteria and preserve the efficacy of existing antibiotics. “One Health” is a concept of global health that emphasised the inter-relation or inter-connection of the health of humans to that of animals (pets, livestock and wild) and the environment. Among bacterial pathogens, staphylococci have been used as suitable models for “One Health” studies, as certain species and clones have been shown to “jump” across the three ecosystems of concern.

*Staphylococcus aureus* (*S. aureus*) is generally a commensal and could be an opportunistic pathogen that causes a wide variety of infectious diseases in humans and animals. This microorganism has a high impact on the general ecosystem, public health and livestock production [1]. AMR, virulence and host adaptation systems in *S. aureus* are of crucial public health concern in livestock, pets and wild animals as they can act as intermittent carriers or reservoirs of zoonoses [1]. Since the last decade, there is an increasing interest but little information about the global prevalence of methicillin-resistant *S. aureus* (MRSA) isolates in wild animals, despite being considered as potential reservoirs or vehicles for transmission [2].

The inter-habitat traversing and the frequent contact between wild animals, livestock and the indirect contact with humans can increase bacterial transmission and often promote the risks of colonisation and infections in humans and animals [3,4,5]. Antimicrobial-resistant bacteria spread by anthropogenic sources, such as industrial and domestic wastewater effluents, agricultural runoff and garbage, have been suspected to be the primary link to wild animals [5,6]. Once certain bacteria get transferred to wild animals, they can be responsible for the spread of many AMR genes, epidemic clones and mobile genetic elements [5,6]. Consequently, these underscore the need for the implementation of control measures against the spread of bacteria across ecosystems to limit the global emergence of novel AMR traits in the future.

MRSA is often multi-drug resistant (MDR), especially to most of the beta-lactam antibiotics (except some new cephalosporins, such as ceftaroline and ceftobiprole) as a result of the synthesis of a modified penicillin-binding protein 2 (PBP2a/c), encoded by the *mec* genes included in the staphylococcal cassette chromosome *mec* (SCC*mec*) [7]. The SCC*mec* are considered mobile genetic elements that could harbour AMR genes other than the *mec* [7]. The *mecA* gene (encoding PBP2a) has been detected in most MRSA isolates of animals, humans and the environment [8]. However, the origin and reservoir host of the *mecC* gene (which encodes the PBP2c) in MRSA has not been fully determined. Initially, *mecC* was related with livestock associated (LA)-MRSA; however, it's continued and increased detection in wild animals indicates that *mecC*-MRSA is primarily associated with wildlife [9]. This suggests that *mecC*-MRSA could be considered as wildlife-associated MRSA (WA-MRSA) [8,9].

In addition to the ability of *S. aureus* to acquire antimicrobial resistance determinants, this species contains an extensive number of virulence factors, ranging from the bacterial cell wall components to different exoproteins (cytotoxins, hemolysins, pyrogenic toxin superantigens and exfoliatins). Among them, deserving special attention, the Panton-Valentine Leukocidin (encoded by *luk*-S/F-PV) that produce the destruction of leukocytes causing necrotising pneumonia, skin and soft tissue infections. Moreover, the toxin that has been associated with toxic shock syndrome (encoded by *tst*) and exfoliative toxins (encoded by *eta, etb, etd* and *etd2*) produce skin lesions, as they prevent cell adhesion between keratinocytes [10]. These virulence factors contribute to the ability of this *S. aureus* to establish and maintain infectious diseases in humans and animals.

It has been demonstrated that *S. aureus* can adapt to humans and different animal species. However, some genes can facilitate its adaptation to a specific host. Thus, it has been observed that the presence of some genes (*scn, chp, sak, sea/sep*) allows the bacterium to survive in humans through the ability to evade the human innate immune response. These groups of genes are collectively known as IEC (immune evasion cluster). Among them, the *scn* gene, which encodes the Staphylococcal Complement Inhibitor (SCIN) is present in all IEC types and considered a good marker for the presence of the IEC system [11].

The ability of *S. aureus* to colonise and adapt to various animal hosts makes it a well-studied pathogen. Moreover, the study of *S. aureus* molecular ecology has provided great insight into the ability of certain bacteria clones to exhibit “inter-species animal jump or spill-over”. While some clonal complexes (CCs) of MRSA appear to be associated with certain animal hosts (for instance, MRSA-CC398 in pigs or MRSA-CC5 in poultry), other CCs such as CC1 and CC130 seem to have a wide host spectrum [12]. Among them, the MRSA-CC130, which was first linked to bovine mastitis, is very relevant in animal health and animal products [13]; however, more recently, it has been found repeatedly in wild animals and very less frequently in humans and the environment (river water), and it is largely associated with the *mecC* mechanism of methicillin resistance [11]. These special clones of MRSA (such as CC398 and CC130) could be transmitted across different “One Health” domains, which requires monitoring and vigilance.

Wild animals could discharge nasal and oral (saliva) secretions [14] which may constitute important transient or persistent vectors of MRSA transmission to humans and other animal species [15], depending on the extent of urban or farmland proximity and interaction [16]. Anatomically, the nasal cavity of animals has a short connection route to the trachea, similarly, the oral (buccal) cavity to the pharynx [16]. Hence, it is expected that microbes in nasal and oral cavities readily have access to the trachea and pharynx [16].

In this study, we determined the pooled prevalence of nasal, tracheal and/or oral (NTO) carriage of *S. aureus* and MRSA in wild animals, with a special focus on the mechanisms of methicillin resistance (*mecA/mecC)* in MRSA isolates, as well as the frequency of MRSA and MSSA of the lineages CC398 and CC130. Furthermore, the genetic lineages of *S. aureus* isolates carrying relevant virulence genes (*tst, eta, etb, lukS/F-PV* and *scn*) from eligible studies were also systematically reviewed. This study aims to comprehensively summarise and consolidate the literature on the ecology and molecular epidemiology of NTO carriage of *S. aureus* and MRSA in wild animals.

## 2. Methodology

### 2.1. Study Design

Based on the guidelines of Preferred Reporting Items for Systematic reviews and Meta-analyses (PRISMA) (http://prisma-statement.org/PRISMAstatement/checklist.aspx, accessed on 20 August 2021), this systematic review was developed and executed on cross-sectional studies that reported S. aureus, MRSA, MSSA in the nasal, tracheal and oral cavities of wild animals. Special focus was given to *mecA*- and *mecC*-MRSA in wild animals as well as to the prevalence of CC398 and CC130 among MRSA and MSSA isolates from tested wild animals.

### 2.2. Articles Search Strategy

Appropriate and eligible articles published (in English) between 1 January 2011 to 30 August 2021 were searched from bibliographic databases such as PubMed, Scopus, Google Scholar, SciElo and Web of Science.

### 2.3. Inclusion Criteria

Original articles and short communications articles that provided sufficient data about the prevalence of “*S. aureus* nasal, oral or tracheal carriage”, “MRSA carriage”, “MSSA carriage” and “molecular typing” in all categories of wild (free-living) animals were selected and extensively reviewed. Specifically, keywords were carefully selected from the Medical Subjects Headings (MeSH) of the US National Library of Medicine (https://www.ncbi.nlm.nih.gov/mesh/, accessed on 20 August 2021). These included “wild animals”, “MRSA-CC398”, “*mecC*-MRSA”, “livestock-associated MRSA”, “nasal carriage” and “bacterial zoonosis”. For this systematic review, four groups of animals were established with the following considerations:

1. Wild mammals (WM) are comprised of wild boars, red deer, Iberian ibex, deer, lynx, wild rabbits, hedgehogs, European mouflons, red foxes, common genets, bats, shrews and mustelids (otters, European badgers, beech martens, American minks and least weasels), among others. Rodents and primates were excluded from this group. This category of wild mammals has almost absolute confinement to the wildlife. However, it is hypothesised that these animals could contract MRSA from the predation of infected rodents and, in turn, spread them to humans who hunt wild animals. This is particularly possible in geographical locations with abundant forests and poor or no wildlife anti-poaching laws.
2. Wild birds (WB) are comprised of storks, vultures and other birds that are naturally found in the wild.
3. Non-human primates (NHP) are comprised of chimpanzees, monkeys, gorillas, lemurs and apes. These mammals have significant physiological and microbiota similarities to those found in humans.
4. Wild rodents (WR) are comprised of mice and rats, both those confined to forests and those with proximity to human settlements and agricultural farms. These animals are also mammals (small) and originate from bushes or the wild. Importantly, it is expected that they could frequently relocate and transverse into human settlements, households, farms and vice versa. Hence, they are separated from other mammals.

### 2.4. Exclusion Criteria

(i) Studies that contained duplicate data or were overlapping articles, (ii) reviews and conference abstracts, (iii) articles that included fewer than 10 animals, (iv) studies on animals in the zoo and captivity, (v) studies on dead animals before sample collection as the time and cause of death is not certain. Moreover, dead animals might undergo some level of putrefaction that could encourage bacterial growth; thus, (vi) studies on skin, faecal and other animal samples were excluded.

### 2.5. Data Extraction

The following information was extracted when possible: authors, study design, study setting or location, the number of *S. aureus* isolates and proportion of MRSA and/or MSSA isolates, type of specimen, laboratory method employed for detection, antimicrobial susceptibility phenotypes and corresponding genotypes and molecular types of the *S. aureus* isolates.

Finally, 33 full texts were included because they were the only available articles that directly focused on the distribution pattern of the *S. aureus*, MRSA, genetic lineages, AMR phenotypes and genotypes and/or virulence genes in NTO cavities of wild animals.

### 2.6. Statistical Analysis

The pooled prevalence of NTO carriage of *S. aureus*, MRSA and MSSA were calculated. MetaXL Version 5.3 (EpiGear International, Queensland, Australia) was used for all statistical analyses. Pooled prevalence analysis was based on combining the results of multiple cross-sectional studies. Specifically, it involved dividing the mean of the sum population of wild animals with *S. aureus* or MRSA NTO carriage by the total studied population in a homogeneous (specific) animal group.

Where possible, an analysis of pooled prevalence was carried out using the random-effects model. Moreover, the pooled rates of nasal carriage by CC398, CC1, CC130 *S. aureus* isolates (MRSA or MSSA) were calculated using the articles in which molecular characterisation (typing) was performed. During the univariate logistic analysis, the choice for the wild mammals’ group as the referent for comparison with other groups was conducted arbitrarily.

## 3. Main Findings

### 3.1. The Pooled Prevalence of S. aureus and MRSA Isolates

**Figure 1:**
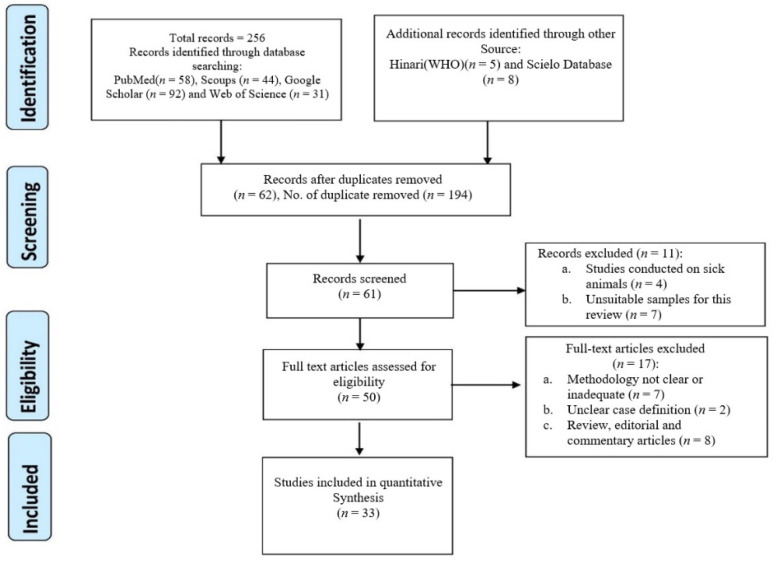
Identification and selection flowchart of articles on NTO staphylococci carriage in wild animals.

Of the 33 eligible and analysed studies (Figure 1), 6, 3, 2 and 22 were from Africa, America, Asia and Europe, respectively [5,12,17,18,19,20,21,22,23,24,25,26,27,28,29,30,31,32,33,34,35,36,37,38,39,40,41,42,43,44,45,46,47]; none were reported in Australia and the Pacific regions. Supplementary Table S1 shows the characteristics and data of the 33 eligible studies, with the indication of the country, type of animals (divided into four groups: wild mammals (WM), non-human primates (NHP), wild birds (WB) and wild rodents (WR)), number of animals tested, number of *S. aureus* and MRSA obtained and the AMR and virulence profiles. The pooled prevalence of NTO carriage of *S. aureus* and MRSA was 18.5% (range: 0–100%) and 2.1% (range: 0.0–63.9%), respectively (Table 1).

**Figure 2:**
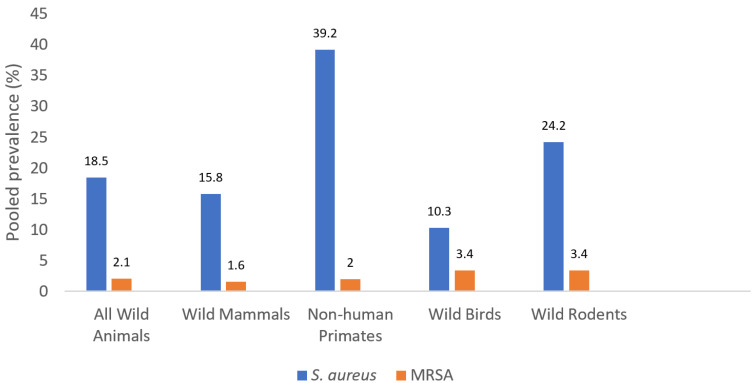
The pooled prevalence of *S. aureus* and MRSA NTO carriage among wild animal groups.

The pooled prevalence of *S. aureus*/MRSA in WM, NHP, WB and WR was: 15.8/1.6, 32.9/2.0, 10.3/3.4 and 24.2/3.4%, respectively (Figure 2, Table 1). There were significant associations between wild animal types and the prevalence of *S. aureus* and MRSA (*p* < 0.05), except in the case of MRSA in WM and NHP (*p* = 0.578) (Table 1). In this sense, the prevalence of MRSA among WB and WR was higher than the one of WM. Moreover, WB had a significantly higher prevalence of MRSA when compared to other wild animals put together (*p* = 0.019) (Table 1).

### 3.2. Prevalence of mecC-MRSA Isolates and Specific Genetic Lineages (CC398, CC130) in the Four Groups of Wild Animals

**Figure 3:**
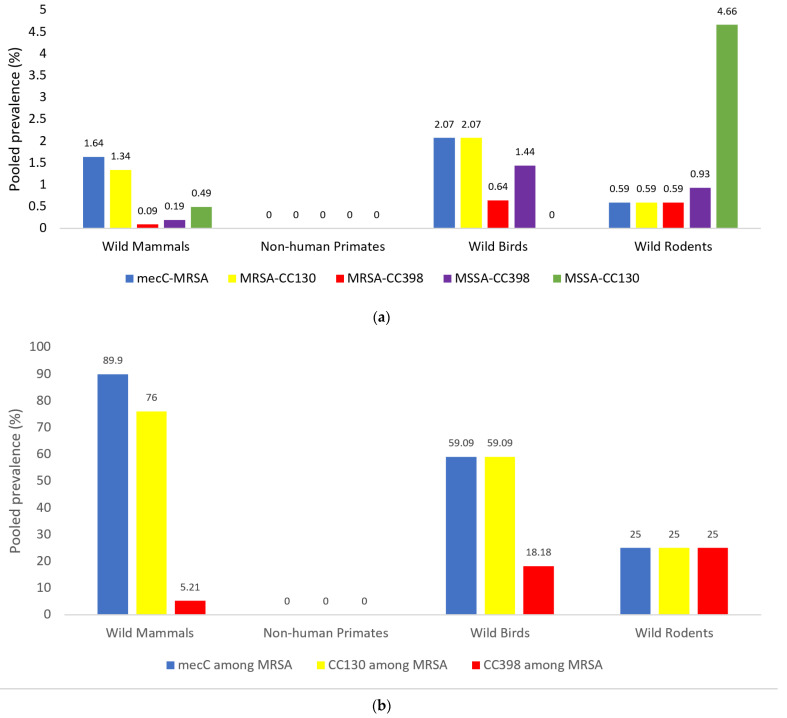
(**a**) The pooled prevalence of *mecC*-MRSA, MRSA-CC130, MRSA-CC398, MSSA-CC398 and MSSA-CC130 among *S. aureus* (MRSA and MSSA) isolates in wild animals of the four studied groups analysed. (**b**) Pooled prevalence rates of *mecC*-positive, CC130 and CC398 isolates among MRSA isolates in wild animals from the four studied groups of wild animals. **Note:** The number of studies per group was as follows: wild mammals (10), non-human primates (4), wild birds (6), wild rodents (4). Some studies recruited more than one animal group.

Figure 3a shows the pooled prevalence of *mecC*-MRSA, MRSA-CC398, MRSA-CC130, MSSA-CC130 and MSSA-CC398 in the four groups of wild animals analysed. In addition, Figure 3b shows the prevalence of the *mecC* gene as well as of CC398 and CC130 lineages among the MRSA isolates obtained from the four studied groups of wild animals (using the articles in which genetic lineages are studied). As it is shown, *mecC*-MRSA has been reported in WM, WB and WR in low percentages (1.64, 2.07 and 0.59%, respectively) (Figure 3a); nevertheless, the mechanism *mecC* is predominant among the MRSA isolates recovered from WM (89.9%) and WB (59.1%), with relatively lower pooled prevalence in WR (25.0%) (Figure 3b). The pooled prevalence of MRSA-CC130 among WM, WB and WR groups was 76.0, 59.1 and 25.0%, respectively (Figure 3b). These corresponded to data obtained for *mecC*-MRSA because most of the *mecC*-MRSA belonged to this genetic lineage (except for some *mecC* isolates of the WM group).

The prevalence of MRSA-CC398 was higher in WB (0.64%) and WR (0.59%), in relation to WM (0.09%) (Figure 3a). If we consider the MRSA isolates of wild animals, the CC398 clone was detected in 25.0% of MRSA isolates of WR, 18.18% of WB and 5.21% of WM (Figure 3b).

In relation to MSSA-CC398 isolate, they were detected among WB (1.44%), WR (0.93%) and WM (0.19%), but not among NHP. Moreover, MSSA-CC130 was only reported in WR (4.66%) and WM (0.49%) (Figure 3a). MRSA-CC398 and MRSA-CC130 were mostly reported in wild animals of the European countries and China (Supplementary Figure S1).

### 3.3. Characteristics of mecC MRSA Isolates from NTO Cavities of Wild Animals

The *mecC*-MRSA isolates were detected in ten of the eligible studies related to NTO-carriage, with a total of 106 isolates (Table 2). The *mecC*-positive isolates were in most cases of the clonal complex CC130 (ST130, ST1945, ST3061, ST1583), although isolates of CC2361 and ST2620 lineages were also reported among wild hedgehogs and European otters, respectively [31,34] (Supplementary Table S2), The predominant *spa*-types among the *mecC* -positive isolates were t843 (55.5%) and t1535 (15.3%), both associated with CC130. However, 10 other *spa*-types were detected in the remaining *mecC*-positive isolates: (a) t3256, t10751, t10513, t10893 and t11015 associated with CC130, (b) t4335, associated with CC2620 and (c) t978, t3391, t9111 and t15312 associated with CC2361 (Supplementary Table S2).

Out of the 10 studies on *mecC*-MRSA, the IEC system was analysed in 8 studies with a total of 66 isolates included. Only 3 of these studies reported the presence of IEC-positive isolate, which corresponded to 18 isolates of the 66 tested (27.3%); they were of the *spa*-types t843 (*n* = 8) and t1535 (*n* = 10) (Table 2), and all were IEC-type E; they were obtained from red deer, vultures and magpies of Spain and wild rats of Portugal [17,19,38].

Besides the penicillin, oxacillin and cefoxitin resistance, most of the *mecC*-MRSA strains were susceptible to all the non-beta-lactam antimicrobials tested (101/106, 95.6%) (Table 2). Only one study reported the detection of a few *mecC*-MRSA isolates that were resistant to ciprofloxacin, erythromycin, clindamycin, gentamicin, tetracycline and/or kanamycin, although the mechanisms implicated were not evaluated [34] (Table 2). The *etd2* was detected in the 12 *mecC*-MRSA isolates in which this gene was analysed (Table 2).

### 3.4. Characteristics of S. aureus-CC398 Isolates Detected from NTO Cavities of Wild Animals

The MRSA-CC398 isolates were detected in seven studies among NTO samples of wild animals (*n* = 14 isolates) and corresponded to the sequence types ST398 and ST1232 and the *spa* types t011 (60% of isolates), as well as to t899, t034, t1451, t4552 and t2582 (Supplementary Table S2 and Table 3). In the case of MSSA-CC398 (*n* = 19 isolate), the predominant *spa*-types were t571 and t1451 (68.2%), but t034, t6606 and t3625 were also reported.

Most of the MRSA-CC398 isolates characterised were IEC-negative (6/9, of *spa*-types t011, t2582 and t4652), although some IEC-positive isolates were also reported among wild boar (*spa*-type t899-IEC-type B) or rural rodents (*spa*-t011-IEC type A and *spa*-type t034-IEC-type E) (Table 3). In relation to MSSA-CC398, a total of 15 isolates were characterised for the IEC system and most of them were IEC-type C (11/15, of *spa*-types t571, t1535, t3625 and t6606), although some few IEC-negative isolates were also found in one study (t1451, t571) (Table 3).

Most MRSA-CC398 isolates showed tetracycline resistance (80%). Nevertheless, three MRSA-CC398 isolates obtained from wild rodents in China were tetracycline-susceptible (*spa*-types t4652 and t2582) and, interestingly, one isolate of this study carried the genes of the Panton-Valentine Leukocidin (*spa*-t034 and IEC-type E) [39]. In relation to the antibiotic resistance profile of the MSSA-CC398 isolates, this was determined in 16 of these isolates and erythromycin resistance was found in 10 isolates carrying the *ermT* gene in 9 of them. Phenotype of complete susceptibility to all the tested antibiotics was identified in 5 of the 16 MSSA-CC398 isolates (Table 3). The MSSA-CC398 isolates were detected in wild birds and rodents (Supplementary Table S2). None of the studies on NHP reported the detection of the *mecC*-MRSA, MRSA-CC130, or MRSA-CC398 (Figure 3a,b).

### 3.5. Characteristics of Other S. aureus Lineages Detected from NTO Cavities of Wild Animals

In addition to the MRSA-CC130 (and other *mecC*-MRSA isolates), MSSA-CC130 isolates (mostly of *spa* types t843 and t1535) were reported (Supplementary Table S2). In addition, other clonal complexes of MRSA, such as the CC5, CC88 and CC133, were found in NTO *S. aureus* isolated from various wild animals (Supplementary Table S2).

MRSA isolates of the genetic lineage CC1 were only reported among WM, although with a low pooled prevalence (0.04%), representing 2.1% of total MRSA isolates in this group of wild animals. Specifically, the MRSA-t127-CC1 clone was reported in two studies of wild mammals [21,23] with a pooled prevalence of 0.037%. Conversely, the MSSA-t127-CC1 was detected from four studies on WM and NHP [5,25,45,46] (Supplementary Table S2).

### 3.6. Antibiotic Resistance and Virulence Genes Detected from S. aureus of NTO Cavities of Wild Animals

Penicillin resistance and the *blaZ* gene were the most predominant traits of AMR in both MSSA and MRSA isolated from NTO cavities of wild animals (reported in 13 studies). Other antibiotic resistance genes (*fexA, str, fosB, sdrM, aacC-aphD, erm(C), aph(30)-IIIa, tetM*, *tetK*, and *aac(6′)-Ie-aph(2″)-Ia*) were reported in at least one of the studies which tested for these genes (Supplementary Table S1). Although two studies phenotypically detected resistance to linezolid [18,47], only one study reported the presence of a mutation [A29V] in L22 and an insertion [68KG69] in L4 ribosomal proteins as the molecular mechanism implicated; no linezolid transferable resistance genes were detected in these studies (Supplementary Table S1).

Genes associated with several virulence factors, including leukotoxins and enterotoxins, were reported in some of the reviewed and eligible studies (Table 4). The Panton-Valentine Leucocidin (PVL) gene, *luk-S/F-PV,* was detected in 4 of 33 studies of NHP and WR (5 isolates); PVL-positive isolates detected corresponded to the clonal complexes CC398 (1 MRSA), CC5 (1 MRSA) or CC22 (3 MRSA) (Table 4). Among all eligible studies, eight isolates (five MRSA and three MSSA, of lineages CC5, CC22, CC30 and CC522) were positive for the *tst* gene, encoding the toxic shock syndrome toxin (TSST), and they were obtained in all four studied groups of animals (Table 4). Moreover, several enterotoxin genes (*sea, seb, sed, sec* and *sep*) and exfoliative toxin genes (*eta, etb, etd2*) were detected in five studies. Some studies detected genes encoding other virulence factors, such as *hla* and *hld* (haemolysins).

**Figure 4:**
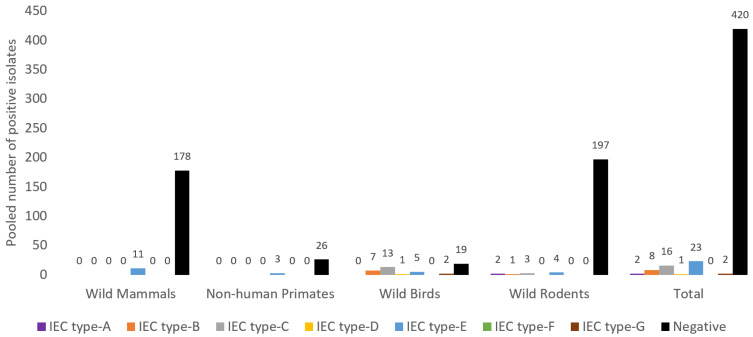
Immune evasion cluster (IEC) type distribution in *S. aureus* isolates from wild animal groups analysed in this study. Note: There were four studies from rodents, one from NHP, two from wild birds and six from wild mammals with IEC analysis (data extracted from Table 2, Table 3 and Table 4).

Out of the total of 472 *S. aureus* isolates from NTO cavities of wild animals tested for the IEC system, 52 were positive (11.0%) and the remaining were IEC-negative (*n* = 420, 89.0%) (Figure 4). Considering the different groups of animals, WB had the highest frequency of IEC-positive isolates (*n* = 28, 59.6%), then NHP (*n* = 3, 10.3%), WM (*n* = 11, 5.8%) and least in WR (*n* = 10, 4.8%). Moreover, the IEC-type E was the most frequently detected among the IEC-positive isolates (44.2%), representing 4.9% of the *S. aureus* IEC-tested isolates (Figure 4).

## 4. Discussion

The human-animal-environment interface (One Health) approach is very fundamental to addressing the threat of AMR, its dissemination and the risks to public health. Although “One Health” research is not yet a priority of many countries, it provides significant data to better understand the global health of humans, animals and their environment [48].

The nasal and oral microbiome ecology has attracted a lot of interest in understanding the scope of AMR, especially in migratory birds. Migratory birds are a type of wild bird that can move a very long distance across several countries. To the best of our knowledge, this is the first comprehensive synthetic and systematic review on the NTO *S. aureus* and MRSA carriage in free-living wild animals. This article provides the NTO *S. aureus* carriage prevalence and pattern across the four major groups of wild animals throughout all the continents of the world. Previous work by Silva et al. [49] was a narrative review focused on the European continent and it dwelled on all types of animal samples (such as skin, faeces and rectal swabs that may have a significant risk of *S. aureus* infection instead of colonisation).

With a global pooled prevalence of 18.5% *S. aureus* NTO carriage in wild animals detected in our review, it can be inferred that this value is slightly higher than those reported in systematic reviews on healthy humans without occupational risk of colonisation (15.9%) and companion animals (17.5%) [50,51]. However, the prevalence of *S. aureus* NTO carriage greatly varies with the category of wild animals and the highest pooled *S. aureus* prevalence was obtained from NHP (32.9%) and least in wild birds (10.3%) (Table 1). The NHP are the closest to humans in respect of microbiota and other physiological compositions. As such, they are expected to have a relatively high rate of *S. aureus* NTO carriage.

Few eligible cross-sectional studies on *S. aureus*/MRSA NTO carriage in primates have been published [41,42,43,44,45,46,47], and most of them have been performed in the African continent. Some other studies were carried out on captive primates for research, breeding and zoological facilities, but these studies were excluded in this review due to the perceived eventual possibility of contracting *S. aureus* from humans as they interact with the primates during day-to-day feeding activities. Conversely, wild birds had the least prevalence of *S. aureus* NTO carriage (10.3%). This is relatively low when compared to other categories of wild animals. The reason for this low data has not been fully elucidated. However, it appeared that *S. aureus* is not often the staphylococcal species associated with the nasotracheal carriage in wild birds (excluding birds of prey) [52]. Moreover, it could be that most of the studied birds had a feeding lifestyle that seldom allows *S. aureus* carriage, as in the case of birds that feed in the natural or semi-natural environment as opposed to those that feed close to landfills [37].

In the study of Ruiz-Ripa et al. [52], about 48.8% of storks showed *S. sciuri* tracheal carriage. However, Gómez et al. [37] reported as high as 34.8% *S. aureus* carriage in white stork nestlings exposed to human residues. So, human residue/garbage (that could be contaminated by *S. aureus*) serves as the major source of food for wild birds (mostly aerial and arboreal), especially migratory birds (such as storks). Conversely, birds of prey such as vultures have shown relatively lesser tracheal *S. aureus* carriage (4.6%) than storks [38]. This difference could be due to variation in feeding habits and food preferences by the birds at the time of sample collections. Moreover, some wild birds feed on dead animal carcases (such as wild boars) that could be colonised by certain *S. aureus* clones. This could be one of the reasons certain wild birds often carry *S. aureus*-CC398 (LA-MRSA) clones mainly adapted to pigs and wild boars [12,25]. Nevertheless, wild birds (especially the migratory ones) can carry pathogens over long distances, thus facilitating pathogen dissemination among human and animal populations [53].

Based on our systematic review of pooled data, pooled NTO MRSA carriage on wild animals was low (2.1%), although few differences were observed depending on the group of animals tested, with slightly higher rates detected in WB and WR (3.4% each) and lower in WM (1.6%) or NHP (2.0%) (Table 1). It is important to remark that there were few heterogeneous studies which make it difficult to reliably assess the statistical differences across the wild animal groups.

**Figure 5:**
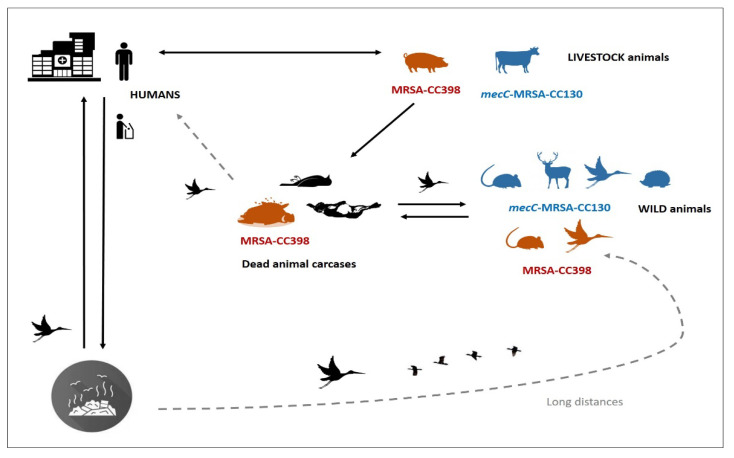
Transmission cycle of special MRSA clones across humans, animals (livestock and wild) and the environment (such as landfills and hospitals). Note: In the silhouettes with colours, the animals in which MRSA-CC398 (red) and *mecC*-MRSA-CC130 (blue) isolates have been detected in high prevalence were illustrated.

Considering the studies in which the mechanisms of methicillin resistance (*mecA* or *mecC* genes) or the genetic lineages of MRSA isolates were analysed, the *mecC*-MRSA was preferentially detected in WB (2.07% of animals tested) and in wild mammals (1.64%), with lower prevalence in wild rodents (0.59%) and no detection in NHP studies. Moreover, the MRSA-*mecA*-CC398 lineage was detected more frequently among wild birds and wild rodents (0.59–0.64%) and lower in wild mammals (0.09%), with no detection on NHP. Interestingly, most MRSA isolates of wild mammals (95%) and wild birds (77%) and 50% of those of wild rodents were typed as *mecC*-MRSA (mostly of lineage CC130) or MRSA-CC398. Put together, it seems that wild animals, especially wild mammals/birds, are natural reservoirs of *mecC*-MRSA-CC130 isolates (supporting its consideration as WA-MRSA) and wild rodents/birds are frequent carriers of the MRSA-CC398 clone (Figure 5). The very high prevalence of *mecC*-MRSA (63.6%) among wild hedgehogs reported in Sweden is of special relevance [34].

Diverse *spa*-types have been detected among MRSA-CC398 isolates, although t011 was the predominant one (60%), highly associated with livestock farming [54]. This *spa*-type was the unique one among MRSA-CC398 in wild birds but was detected combined with other *spa* types in MRSA of wild mammals and wild rodents (Supplementary Table S2). It is interesting to remark that MSSA-CC398 was detected in wild birds, wild rodents and wild mammals (0.19–1.44%), of which *spa* types t571 and t1451 were predominant. However, t034, t6606 and t3625 were detected in lesser frequencies [5,17,37].

The *spa* types t843 and t1535 were the predominant ones among *mecC*-MRSA-isolates, although many other *spa* types were detected. These *spa* types were also the most frequently detected in food-producing animals or human *mecC*-MRSA infections [55]. Both lineages, t843/CC130 and t1535/CC130, have also been found among MSSA isolates of wild boar [23,25] and in free-living wild rats [12].

It has been suggested that there might be a mutual exchange of *mecC*-MRSA between livestock and wild animals since it was thought that CC130 originated in ruminants [56]. Most of the *mecC*-MRSA isolates of wild animals included in this review showed susceptibility for non-beta-lactam antibiotics, with a few exceptions (Table 2). This feature was also previously found in *mecC*-MRSA isolates obtained in human infections [55]. Resistance for non-beta-lactam antibiotics was detected in *mecC*-MRSA recovered from wild hedgehogs in Sweden [34].

Another aspect of interest is the presence of the IEC system (associated with human adaptation) in the *mecC*-MRSA isolates. The demonstration of *scn* gene in wild animals can represent *S. aureus* NTO carriage by a human-adapted strain and could suggest reverse-zoonosis (zooanthroponosis). However, the absence of the IEC gene (with the *scn* marker), can denote a non-human strain and represent a key evolutional event [11]. In this respect, most of the *mecC*-MRSA isolates (72.7%) were IEC-negative; nevertheless, 27.3% were IEC-type E positive. These strains presented the *spa*-types t843 and t1535 and were recovered of red deer, vultures, magpies and wild rodents in Spain and Portugal [17,19,38]. As indicated before, the detection of IEC genes often highlights possible human adaptation. However, it has been proposed that IEC-type E might be a conserved feature of ST1945-MRSA isolate as studies from Spain and Portugal reported IEC-type E in *mecC*-MRSA-ST1945 isolates [17,19,38]. In our systematic review, a pooled prevalence of 27.3% *mecC*-MRSA-IEC-type E positive strains was obtained from eight eligible studies on wild animals (Table 2). This value is relatively high and indicates that IEC-positive-*mecC*-MRSA in the general ecosystem deserves to closely be monitored.

Most of the available *mecC*-MRSA articles in which IEC genes were studied in human infections [55,57], livestock and milk samples [58], river water [59] and even other animals’ faecal and skin samples [60] were IEC-negative; nevertheless, the detection of *mecC*-MRSA-ST1945-IEC-positive strain of human origin, that was type E, has been reported [61]. Similarly, Gómez et al. [62] found *mecC*-MRSA-CC130-IEC-type E strains from rat faecal samples. Moreover, one *scn*-positive MSSA-CC130 isolate was reported by Silva et al. [17]. In all studies in which the *etd*2 was analysed in the *mecC*-MRSA isolates, this toxin gene was detected. This gene was found in the genome of all CC130 isolates (both *mecC*-MRSA and MSSA) analysed in one study which suggests that *etd*2 could be intrinsic for this genetic lineage [11].

As expected, most of the MSSA isolates from wild animals had low-level AMR. The great majority of the isolates were susceptible to all tested antibiotics (Supplementary Table S1). This low prevalence of AMR in wild animals could be because these animals do not directly encounter antibiotics and have no evolutionary selective pressure [33]. Although the presence of AMR in wild animals depends on the location where they are found and the category of animals, some studies have identified wild animals with apparently no contact with antibiotics to be colonised with *S. aureus* with certain AMR genes [37,38,47,60]; therefore, MRSA NTO carriage in wild animals may be considered a sentinel of AMR. The most frequently detected AMR in MSSA was for penicillin (Supplementary Table S1). As shown in Table 3, all MRSA-CC398 isolates included in this review showed tetracycline resistance and, when tested, carried the *tetM* gene (and, in many cases, also *tetK*). This phenotypic/genotypic characteristic has been proposed as a marker of the MRSA-CC398 clone in different studies [63,64].

Since its discovery in the early 2000s to date, MRSA-CC398 has consistently been detected in humans with contact with farm animals and in a wide variety of animals (especially in pigs) and their environments. However, lately, the MSSA-CC398 strains have also attracted interest for epidemiological and evolutionary purposes and because MSSA-CC398 strains could be implicated in emergent invasive human infections [65]. In this review, it appears that storks and rodents could be the major wild animal reservoirs of this MSSA genetic lineage (mainly with the *spa* type t571) [17,37]. It is worthy to remark that MSSA-CC398 isolates have been recovered from other animals, such as the aquatic ones [63,66]. From the phylogenetic and evolutionary point of view, the CC398 lineage of *S. aureus* was postulated to have two separate host sub-clades: (a) a livestock associated-clade in which *S. aureus*-CC398 carries the *mecA* and *tetM* genes and lacks the *scn* gene (associated to phage φ3-Sa) and (b) a human associated-clade (MSSA) carrying *scn* (human adaptation gene) but no *tetM* [64]. Various sub-clades have emerged and spread across different animals, animal products (e.g., meat) and countries and continents [54].

The detection of MRSA-CC1 in wild boars and rabbits shows that this clone has a clear potential of establishment and spread in the wild and perhaps transmission into livestock farms and the urban community. Aside from these genetic lineages, MSSA-CC425 was also detected in 13.7 and 24.4% of wild boars by Mama et al. [25] and Seinige et al. [33], respectively, and wild birds [38]. Similarly, MSSA-CC1 strains were often reported from the nasal cavities of NHP in sub-Saharan Africa [42,43,46]. ST425/CC425 is a genetic lineage with a pattern of transmission that has been attributed to wild animals NTO colonised through the ingestion of secretions from carnivorous animals (e.g., foxes) [67]. Thus, the report of MSSA-ST425 from the wildlife deserves to be monitored.

Aside from methicillin resistance, resistance to linezolid (one of the last resort antimicrobial agents) was reported in one MRSA-ST2328 isolate of wild boar [18] and two MRSA-ST22/ST88 isolates of monkeys [47]; although transferable linezolid resistance genes were not detected in these isolates, mutation and insertion in L22 and L4 ribosomal proteins, respectively, were detected [47] (Supplementary Table S1). The detection of linezolid-resistant *S. aureus* isolates carrying the ribosomal mutation, which confers a very high resistance level for linezolid, is relevant, although it has no capacity for horizontal transference [68].

The *lukS/F-PV* virulence gene was rarely reported in *S. aureus* of wild animals; however, five studies detected PVL-positive MRSA and MSSA isolates from NHP and WR [39,42,43,46,47]. Interestingly, most of the PVL-positive isolates were detected in MSSA or MRSA in NHP [42,43,46,47], although there is one study performed on urban rodents in China which detected the PVL-positive-MRSA-CC398-t034 isolate [39]. It is worthy to mention that MRSA strains in rats in contact with cattle can be colonised by LA-MRSA [12]. Moreover, the LA-MRSA-PVL positive strains deserve to be meticulously monitored. This suggests that PVL-carrying *S. aureus* derived from NTO cavities of NHP and rodents may play a role as maintenance hosts or vectors for MRSA, which is important to human health. PVL is a significant pore-forming toxin that is often associated with abscesses. *S. aureus* carrying PVL appears to be endemic in humans in sub-Saharan Africa [50]. However, its role in some wild animals (as NHP and urban rodents) needs to be studied in detail. Perhaps, these animals have different selection pressure for PVL-positive *S. aureus* isolates.

Similarly, the *tst* gene encodes the pyrogenic toxin superantigen TSST-1, one of the most important virulence proteins of *S. aureus* that produces limited or systemic infections. This gene is located on staphylococcal pathogenicity islands that facilitate *S. aureus* immunopathogenesis through the secretion of anti-inflammatory chemokines and induction of immunosuppression [69]. The TSST-1 is often mobilised and elaborated with the help of many bacteriophages [70]. The *tst* gene has been detected in MSSA or MRSA isolates in five studies of wild mammals, wild birds and NHP (Table 4).

Despite the comprehensiveness of this article in providing updated data on *S. aureus* and MRSA nasal, oral and tracheal carriage of wild animals, it is necessary to interpret these data with caution, as the pooled prevalence generated from the animal groups and continents may not be the absolute measure of the extent of the genetic lineages, AMR and virulence factors of *S. aureus* in these entities.

## 5. Conclusions

Although the global prevalence of MRSA is low in wild animals, the *mecC*-mediated mechanism was particularly prevalent among MRSA isolates (especially among those of wild mammals and birds). Moreover, the global prevalence of MRSA-CC398 lineage was low in wild animals but its prevalence among MRSA was relatively high, especially in wild birds. The NTO cavities of wild animals are potential vehicles of *S. aureus*/MRSA transmission, but the extent appears to vary according to the animal type and geographic location of studies. Findings from this systematic review showed that wild animals could carry AMR, virulence genes and genetic lineages of human, agricultural and epidemiological importance across the “One Health” domains. Particularly, the reports of *lukS/F-PV, tst* and linezolid resistant carrying MRSA are of great concern. Considering the genetic diversity of MRSA in wild animals, they need to be continuously monitored for effective prevention and control of AMR.

## Figures and Tables

**Table 1:** (**a**) Summary of the pooled global prevalence of *S. aureus* and MRSA NTO carriages in the four studied wild animal groups. (**b**) Comparative prevalence of *S. aureus* and MRSA carriages between wild birds and other wild animals.

(**a**)													
**Study Groups**	**Number of *S. aureus* Studies Included**	**Total Number**		**Pooled *S. aureus* Carriage Rate (%) (Range)**	**OR (95% CI)**	***p* Value**	**Number of MRSA Studies Included**	**Total Number**		**Pooled MRSA Carriage Rate (%) (Range)**	**OR (95% CI)**	***p* Value**	**Total Number of Studies Included ^a^**
		**Animals**	***S. aureus***					**Animals**	**MRSA**				
Wild Mammals (excluding rodents and NHP)	13	3031	479	15.8 (0.0–36.9)	Referent	Referent	17	6110	99	1.6 (0.0–63.6)	Referent	Referent	18
Wild Rodents	4	856	207	24.2 (15.3–41.0)	1.69 (1.41–2.04)	<0.0001	5	1452	49	3.4 (0.3–4.7)	2.12 (1.49–3.00)	<0.0001	5
Non-human Primates	7	403	158	39.2 (0.0–100.0)	3.44 (2.78–4.29)	<0.0001	7	403	8	2.0 (0.0–26.7)	1.23 (0.59–2.55)	0.578	7
Wild Birds	5	586	60	10.3 (5.0–34.8)	0.61 (0.46–0.81)	0.0006	6	626	21	3.4 (0.0–4.0)	2.11 (1.31–3.40)	0.002	6
Total Wild Animals	29	4876	905	18.5 (0.0–100)	NA	NA	35 ^a^	8601	177	2.1 (0.0–63.9)	NA	NA	36 ^a^
(**b**)													
**Study Groups**	**Number of *S. aureus* Studies Included**	**Total Number**		**Pooled *S. aureus* Carriage Rate (%) (Range)**	**OR (95% CI)**	***p* Value**	**Number of MRSA Studies Included**	**Total Number**		**Pooled MRSA Carriage Rate (%) (Range)**	**OR (95% CI)**	***p* Value**	**Total Number of Studies Included ^a^**
		**Animals**	***S. aureus***					**Animals**	**MRSA**				
Wild Animals (excluding wild birds)	24	4290	844	19.7 (0.0–100.0)	Referent	Referent	29	7965	156	1.9 (0.0–63.6)	Referent	Referent	30
Wild Birds	5	586	60	10.3 (5.0–34.8)	0.46 (0.35–0.61)	<0.0001	6	626	21	3.4 (0.0–4.0)	1.74 (1.09–2.76)	0.019	6

^a^ Studies that analyse either *S. aureus*, MRSA or both. **Key:** NA = not applicable; OR = odd ratio; CI = confidence interval; Significant association and effect size of *S. aureus*, MRSA and types of the wild animal groups determined by bivariate logistic regression (*p* < 0.05).

**Table 2:** Genetic lineages, AMR, virulence genes and IEC system in *mecC*-MRSA isolates detected in NTO *S. aureus* carriage studies in wild animals.

Reference	Animal Species (Location)	No. of Animals Tested	No. of *S. aureus*	No. of *mecC*-MRSA (% Colonised Animals)	*spa*-type/ST/CC (Number of Isolates)	AMR Phenotype for Non-Beta-Lactams of *mecC*-MRSA	IEC-type in *mec*C-MRSA (Number Isolates, *spa*)	**Other Virulence Genes in *mecC*-MRSA Isolates (Number Strains)**
[12]	Wild free-living rodents (Germany)	145	37	1 (0.7)	t843 (1)/CC130 (1)	Susceptible (all)	IEC-negative (1)	NT
[17]	Wild rodents (Portugal)	204	38	3 (1.5)	t1525 (3)/ST1945 (3)/ CC130 (3)	Susceptible (all)	IEC-E (3, t1535)	Negative (for *lukS/F-PV, hla, hlb, eta, etb, tst)* (3)
[19]	Red deer (Spain)	65	16	11 (16.9)	t843 (4), t1535 (7)/CC130 (11)	Susceptible (all)	IEC-E (11, t843, t1535)	*etd2* (11)
[22]	Wild rodents and shrews (Germany, Czech and France)	295	45	1 (0.3)	t843 (1)/CC130 (1)	Susceptible (all)	IEC-negative (1)	Negative (for *lukS/F-PV*, *sea*-*seu*, *tst*, *eta*, *etd*) (1)
[23]	European hedgehog, European rabbit, red deer, wild boar, European mouflon (Spain)	103	23	3 (2.9)	t843 (3)/ST130 (3)/CC130 (3)	Susceptible (all)	IEC-negative (3)	*seg* (1), *seh* (1). Negative for *lukS/F-PV, tst, eta, etb*, and 18 enterotoxin genes
[29]	Rabbit and hare (Spain)	363	70	34 (9.3)	ST1945 (33), ST5823 (1)/CC130 (34)	Susceptible (all)	IEC-negative (34)	NT
[31]	European brown hare, European otter, European hedgehog, Eurasian lynx (Germany)	40	5	5 (12.5)	t843 (2), t10513 (1), t3256 (1), t4335 (1)/ST2620 (1). ST130 (4)/CC130 (5)	NT	NT	NT
[34]	Wild hedgehog (Sweden)	55	35	35 (63.6)	t843 (17), t10751, t978 (3), t9111 (3), t15312 (4), t3391 (5), t10893 (1), t11015 (1)/CC130 (20), CC2361 (15)	CIP (5), CLI (6), ERY (5), GEN (7), KAN (5), TET (2)	NT	NT
[37]	Stork (Spain)	92	32	1 (1.1)	t843 (1)/ST3061 (1)/CC130 (1)	Susceptible (all)	IEC-negative (1)	*etd2* (1)
[38]	Cinereous vulture and magpie (Spain)	324	15	12 (3.7)	t843 (11), t1535 (1)/CC130 (12)	Susceptible (all)	IEC-E (4, t843) IEC-negative (8, t843 and t1535)	Negative (for *lukS/F-PV, tst, eta, etb*, and *etd*) (12)

**Key:** NT: not tested; CLI: Clindamycin; CIP: Ciprofloxacin; ERY: Erythromycin; GEN: Gentamicin; KAN: Kanamycin; TET: Tetracycline.

**Table 3:** Genetic lineages, AMR, virulence genes and IEC system in MRSA- and MSSA-CC398 isolates detected in NTO carriage studies in wild animals.

Reference	Animal Species (Location)	No. of MRSA-CC398	*spa*/ST of MRSA-CC398 (Number of Strains)	IEC-type (Number of Strains) in MRSA-CC398	AMR Phenotypes/Genes (Number Strains) of MRSA-CC398	Other Virulence Genes (Number Strains) of MRSA-CC398	No. of MSSA-CC398 (%)	*spa*/ST of MSSA-CC398 (Number of Strains)	IEC-type (Number of Strains) in MSSA-CC398	AMR Phenotypes/Genes (Number Strains) in MSSA-CC398	Other Virulence Genes (Number Strains) in MSSA-CC398
[5]	Iberian ibex, red deer and wild boars (Spain)	0	NA	NA	NA	NA	3	t034 (2), t571 (1)/ST398/CC398	NT	TET (3)	NT
[17]	Wild rodents (Portugal)	0	NA	NA	NA	NA	6	t1451 (5), t571 (1)/ST398 (4), ST5926 (2)	IEC-C (2), IEC-negative (4)	Susceptible (all)	*hld* (all)
[18]	Wild boars (Portugal)	1	t899/ST398	IEC-B (1)	TET, PEN FOX, OXA, CIP/*mecA*	NT	0	NA	NA	NA	NA
[21] ^a^	Wild mammals (Spain)	3	t011 (2), t1451 (1)/ST398 (3)	NT	TET (3), CIP (2), ERY (1), CLI (1)	NT	0	NA	NA	NA	NA
[21] ^a^	Eurasian griffon vulture (Spain)	2	t011 (2)/ST398 (2)	NT	TET (2), CIP (1), ERY (1), CLI (1)	NT	0	NA	NA	NA	NA
[25]	Wild boar (Spain)	1	t011/ST398 (1)	IEC-negative (1)	PEN- FOX- TET/*blaZ, mecA, tet(M), tet(K)*	Negative (for *lukS/F-PV, tst, eta, and etb*) (1)	0	NA	NA	NA	NA
[33]	Wild boar (Germany)	0	NA	NA	NA	NA	1	t571 (1)/ST804 (1)	NT	AMP (1), ERY (1)/*blaZ*	Negative (for *sea, seb, sec, sed, see, she, eta, etb, tst, luk-S/F-PV)* (1)
[37]	Stork (Spain)	1	t011 (1)/ST398 (1)	IEC-negative (1)	PEN, OXA, FOX, TET/*mecA, tetK, tetM*	*cna* (1)	9	t571 (5), t6606 (3), t3625 (1)/ / ST398 (8), ST2377 (1)	IEC-C (9)	PEN (all), ERY (all), CLI (all)/ *blaZ, erm(T)*	*cna* (all)
[38]	Cinereous vulture (Spain)	1	t011 (1)/ST398 (1)	IEC-negative (1)	PEN, FOX, ERI, CLI, TET/*mecA, blaZ, erm(C), vga(A), tetK, tetM*	Negative (for *lukS/F-PV, tst, eta, etb*, and *etd*) (1)	0	NA	NA	NA	NA
[39]	Rodents (China)	5	t034 (1), t011 (1), t4552 (1), t2582 (2)/ST398 (4), ST1232 (1)	IEC-E (1), IEC-A (1) IEC-negative (3)	TET (2), AZM (1), CLI (1)	*lukS/F-PV* (1, *spa* t034)	0	NA	NA	NA	NA

**Key:** NT = not tested; NA: not applicable; ST = sequence type; CC = clonal complex; AMP: Ampicillin; AZM: Azithromycin; CLI: Clindamycin; CIP: Ciprofloxacin;; ERY: Erythromycin; FOX: Cefoxitin; OXA: Oxacillin; PEN: Penicillin; TET: Tetracycline; ^a^ studies on more than one animal group.

**Table 4:** (**a**) Studies in which the TSST-1, PVL and IEC encoding genes were analysed among *S. aureus* isolates. (**b**) Characteristics of *S. aureus* isolates carrying *lukS/F-PV*, *tst* or *eta* virulence genes.

(**a**)									
**Reference**	**Animal Species**	**No. of Animals Tested/*S. aureus*/MRSA**	**No. of *tst* (%) in MRSA**	**No. of *tst* (%) in MSSA**	**No. of *lukS/F-PV* (%) in MRSA**	**No. of *lukS/F-PV* (%) in MSSA**	**No. Strains with IEC (%) in MSSA**	**No. Strains with IEC (%) in MRSA**	**No. of *S. aureus* IEC-Negative (%)**
[12]	Wild free-living rodents	145/37/2	1 (50.0)	0	0	0	0	IEC-E, 1 (50.0)	36 (97.3)
[17]	Wild rodents	208/38/6	0	0	0	0	IEC-E, 1 (3.1)	IEC-E, 3 (50.0)	31 (81.6)
							IEC-C, 2 (6.2)	IEC-A, 1 (16.7)	
[18]	Wild boars	45/15/1	NT	NT	NT	NT	NT	IEC-B, 1 (100.0)	14 (93.3)
[19]	Red deer	65/16/11	0	NT	0	NT	NT	IEC-E, 11 (100.0)	5 (31.3)
[23]	Wild mammals	103/23/4	0	1 (5.3)	0	0	0	0	23 (100.0)
[37]	Storks	92/32/3	0 (0.0)	2 (6.9)	0	0	IEC-B, 6 (20.7)	0	5 (15.6)
							IEC-C, 11 (37.9)		
							IEC-D, 1 (3.4)		
							IEC-G, 9 (31.0)		
[38]	Wild birds	324/15/13	0	0	0	0	IEC-E, 1 (50.0)	IEC-E, 4 (30.8)	10 (66.7)
[39]	Urban rodents	212/87/11	NT	NT	2 (18.2%)	NT	NT	IEC-E, 1 (9.1), IEC-IEC-G, 1 (9.1)	NT
								IEC-B, 1 (9.1)	
								IEC-A, 2 (18.2)	
[42]	NHP	132/15/0	ND	Present but number not specified	Present but number not specified	0	0	NT	NT
[43]	NHP	62/36/0	NT	0	0	10 (27.8)	NT	NT	NT
[46]	NHP	95/58/0	NT	0	0	2 (3.4)	NT	NT	NT
[47]	NHP	59/29/4	3 (75.0)	NT	3 (75.0)	0	0	IEC-E, 3 (75.0)	26 (89.7)
(**b**)									
**Reference**	**Origin of Isolates**	***Spa*-type/ST/CC of Positive Isolates (No. of Isolates)**	**Virulence Gene**	**Methicillin Resistance Phenotype**	**IEC-type (Number of Strains)**				
[12]	Germany/Rodent/Nasal	t684/CC30 (1)	*tst*	MRSA	E (1)				
[23]	Spain/Wild boar/Nasal	t1534/CC522 (1)	*tst*	MSSA	IEC-negative				
[37]	Spain/Storks/Trachea	t012/CC30 (2)	*tst*	MSSA	D (1)				
[47]	Nepal/NHP/Oral	ST22 (3)	*tst*	MRSA	E (3)				
[37]	Spain/Stork/Trachea	t209/CC5 (1)	*eta*	MSSA	B (1)				
[39]	China/Wild Rodents/Nasal	t034/ST1232/CC398 (1)	*luk*-*SF-PV*	MRSA	G (1)				
		t127/ST1/CC5 (1)							
[43]	Zambia and Uganda/NHP/Nasal	ST80 (9)	*luk-SF-PV*	MSSA	NT				
		ST2178 (1)							
[46]	Gabon and Cote d’ Ivoire)/ NHP/Nasal	ST1855 (1)	*luk-SF-PV*	MSSA	NT				
		ST 1928 (1)							
[47]	Nepal/NHP/Oral	ST22 (3)	*luk*-*SF-PV*	MRSA	E (3)				

**Key**: NT = not tested; TSST-1 = toxic shock syndrome toxin-1; IEC = immune evasion cluster; PVL = Panton Valentine Leucocidin. (**b**) NT: not tested; ST: sequence type; CC: clonal complex. **Note**: In the IEC system, the presence of *scn* is found in all IEC types and frequently utilised as the determinant for IEC-positive *S. aureus* isolates. Essentially, the presence of ≥2 of the 5 genes associated with the IEC determines the IEC type of the *S. aureus* isolate. There are seven IEC types (A to G) depending on the combination of *scn, chp, sak, sea/sep genes:* IEC-type A (*sea, sak, chp, scn*), IEC-type B (*sak, chp, scn*), IEC-type C (*chp, scn*), IEC-type D (*sea, sak, scn*), IEC-type E (*sak, scn*), IEC-type F (*sep, sak, chp, scn*) and IEC-type G (*sep, sak, scn*).

## Data Availability

The data used for this systematic review and meta-analysis can be made available upon request through the corresponding author.

## Supplementary Materials

The following are available online at https://www.mdpi.com/article/10.3390/antibiotics10121556/s1, Figure S1: Geographic distribution of MRSA-CC398 and *mec*C-MRSA isolates detected in NTO cavities of wild animals, Table S1: Study characteristics, antimicrobial resistance, and virulence genes of *Staphylococcal aureus* NTO carriages in wild animals, Table S2: Molecular typing reports of *S. aureus* isolated from the naso-tracheo-oral cavity of various free-living wild animals.
