# Supplementary material for: Wild Animals Are Reservoirs and Sentinels of Staphylococcus aureus and MRSA Clones: A Problem with “One Health” Concern
Source: Antibiotics (Basel). 2021 Dec 20;10(12):1556. doi: 10.3390/antibiotics10121556 (PMC8698730; doi:10.3390/antibiotics10121556)
Supplement: Supplementary file 1 [file antibiotics-10-01556-s001.zip › antibiotics-1491616-supplementary-corrected.pdf]

antibiotics

Systematic Review

Wild Animals Are Reservoirs and Sentinels of *Staphylococcus aureus* and MRSA Clones: A Problem with "One Health" Concern

Idris Nasir Abdullahi, Rosa Fernández-Fernández, Guillermo Juárez-Fernández, Sandra Martínez-Álvarez, Paula Eguizabal, Myriam Zarazaga, Carmen Lozano and Carmen Torres *

Area of Biochemistry and Molecular Biology, One-Health Research Group, Universidad de La Rioja, 26006 Logroño, Spain; idris-nasir.abdullahi@unirioja.es (I.N.A.); rosa.fernandez.1995@gmail.com (R.F.-F.); gukle.juarez@gmail.com (G.J.-F.); sandra.martinezal@unirioja.es (S.M.-Á.); paulaeguimar@gmail.com (P.E.); myriam.zarazaga@unirioja.es (M.Z.); carmen.lozano@unirioja.es (C.L.)

* Correspondence: carmen.torres@unirioja.es

Supplementary Materials

Table S1. Study characteristics, antimicrobial resistance, and virulence genes of *Staphylococcal aureus* NTO carriages in wild animals.

Reference	Animal Species (Description)	Country	Number of Animals tested	No. of *S. aureus* positive animals (%)	Total number of MRSA positives in animals (%)	Laboratory detection methods	Antimicrobial resistance phenotypes (Aside from MRSA)	AMR genes	Virulence genes detected
**Wild Mammals (WM, excluding NHP and wild rodents)**									
[5][a]	Wild mammals	Spain	1120	188 (16.7)	0 (0.0)	C/P/M	PEN, SXT, TET, STR	NT	NT
[18]	Wild boars	Portugal	45	15 (33.3)	1 (8.9)	C/P/M	TET, PEN, CLI, GEN, CIP, LZD	*aac(6′)-Ie-aph(2″)-Ia*	IEC type B
[19]	Red deer	Spain	65	16 (24.6)	11 (16.9)	C/P/M	PEN	*blaZ*	IEC type E, *etd2*

[20]	Wild Boars	Germany	114	8 (7.0)	0 (0.0)	C/P/M	Fosfomycin-susceptible	*fosB*	ND
[21][a]	Wild mammals (red deer, Iberian ibex)	Spain	2506	NR	4 (0.01)	C/P/M	TET, CIP, ERY, CLI	NT	NT
[23]	Wild mammals	Spain	103	23 (22.3)	4 (3.9)	C/P/M	PEN, FOX, ERI, CLI, STR	*mecA, mecC, blaZ, erm(C), aph(3′)-IIIa, ant(6)-Ia*	*seh, seg, sei, sem, sen, seo, seu, tst*
[25]	Wild Boar	Spain	371	51 (13.7)	1 (0.3)	C/P/M	PEN, STR, TET	*blaZ*, *tet*(M), *tet*(K), str	*luk*-D, *lukE*
[26]	Wild rabbit	Spain	59	17 (28.8)	NT	C/P/M	NT	NT	NT
[27]	Wild mammals	Spain	103	23 (22.3)	3 (2.9)	C	PEN, MUP, CLI, STR, LZD, TET	NT	NT
[28]	Wild Boars	Germany	577	0 (0.0)	0 (0.0)	C/P/M	ND	ND	ND
[29]	Wild Lagomorphs	Spain	369	70 (18.9)	34 (9.4)	C/P/M	PEN	*blaZ*	ND
[30]	Wild Ungulates	Spain	139	0 (0.0)	0 (0.0)	C/P/M	ND	ND	ND
[31]	Wild mammals	Germany	40	5 (12.5)	5 (12.5)	C/P/M	PEN	*blaZ*	ND
[32]	Wild rabbits	Portugal	77	4 (5.2)	0 (0.0)	C/P/M	PEN	*blaZ*	*hlB*
[33]	Wild boar	Germany	111	41 (36.9)	0 (0.0)	C/P/M	AMP, PEN, ERY	*blaZ*	*she*
[34]	wild hedgehogs	Sweden	55	NR	35 (63.6)	C/P/M	PEN, GEN, CIP, ERY	NT	NT
[35]	Bats	Gabon and Cote d' Ivoire	133	4 (3.0)	0 (0.0)	C/P/M	ND	ND	*hla*
[39][a]	Shrews	China	185	14 (7.6)	1 (0.5)	C/P/M	PEN	*mecA*	ND

**Non-Human Primates (NHP)**									
[41]	Wild primates	Uganda	15	15 (100.0)	4 (28.6) C/P/M		PEN, SXT, TET, RIF	NT	NT
[42]	Wild Primates	Côte d'Ivoire, Gabon, Democratic Republic of Congo, and Senegal	132	15 (11.3)	0 (0.0)	C/P/M	PEN, TET, SXT, ERY	NT	*lukS/F-PV, tst, sea, seb, eta, etb*
[43]	Wild Apes	Zambia and Uganda	62	36 (58.1)	0 (0.0)	C/P/M	PEN, SXT, TET, ERY, CLI	NT	*lukS/F-PV*
[44]	Wild Monkeys	Saint Kitts	21	2 (9.5)	0 (0.0)	C/P/M	ND	ND	ND
[45]	Wild apes and lemur	Madagascar	19	7 (36.8)	0 (0.0)	C/P/M	PEN	ND	ND
[46]	Wild Monkeys	Gabon, Cote d' Ivoire	95	58 (61.1)	0 (0.0)	C/P/M	PEN	*blaZ*	*lukS/F-PV*
[47]	Rhesus macaques	Nepal	59	29 (49.2)	4 (6.8)	C/P/M	ERY, CLI, LZD, TET, GEN	*aacC-aphD, erm(C), blaZ,* L22: [A29V] and L4: insertion [68KG69]	*sak, scn, tst, sec3, seo, sem, sei, sen, seg, ebpS, sdrE, cna, clfA, map, chp, lukS/F-PV, hlgA, hlgB, hld*
**Wild Birds (WB)**									
[5][a]	Wild vulture	Spain	40	2 (5.0)	0 (0.0)	C/P/M	ND	NT	NT
[21][a]	Wild vulture	Spain	40	NR	2 (0.01)	C/P/M	TET, CIP, ERY, CLI	NT	NT
[24]	Wild birds	USA	114	10 (8.8)	3 (2.6)	C/P/M	TET, LEV, ERY, CLI	NT	NT
[36]	Birds of prey	Portugal	16	1 (6.3)	0 (0.00	C/P/M	PEN, TET, CHL	*blaZ*	ND

[37]	Storks	Spain	92	32 (34.8)	3 (3.3)	C/P/M	PEN, CIP, ERY, CLI, CHL, STR	*blaZ, erm(T), tet(M), str, fexA*	*tst, can, eta, etd2*
[38]	Magpies and Cinereous Vultures	Spain	324	15 (4.6)	13 (4.0)	C/P/M	PEN, FOX, ERI, CLI, TET	*blaZ, erm(C), vga(A), tet(K), tet(M)*	IEC type E
**Wild Rodents (WR)**									
[12]	Free living wild rats	Germany and Czech Republic	145	37 (25.5)	2 (13.8)	C/P/M	PEN	NT	*tst*, IEC type E
[17]	Wild Rodents	Portugal	204	38 (18.6)	6 (2.9)	C/P/M	PEN, CIP, ERY, CLI, KAN, GEN	*blaZ, aph(30)-IIIa, ermA*	IEC Types A and E, *hlb, hld*
[22]	Wild rodents and shrews	Germany, Czech and France	298	45 (15.1)	1 (0.3)	C/P/M	NR	*blaZ, fosB, sdrM*	*Seg, egc, LukD, M* and *X*
[39][a]	Rodents	China	212	87 (41.0)	11 (5.2)	C/P/M	PEN, SXT, CIP, TET, GEN	NT	*lukS/F-PV*, IEC types A, B, E, G
[40]	Wild rats	Canada	596	NR	29 (4.9)	C	NT	NT	NT

Key: Detection method: C//P/M: Culture / PCR / Molecular typing
NT: Not tested
NA: Not available (tested but result not published on the article)
ND: Not detected
NR: Not reported in detail
CHL: Chloramphenicol; CLI: Clindamycin; CIP: Ciprofloxacin; ERY: Erythromycin; FOX: Cefoxitin; GEN; Gentamycin; KAN: Kanamycin; LEV: Levofloxacin; LZD: Linezolid; MUP: Mupirocin; OXA: Oxacillin; PEN: Penicillin; STR: Streptomycin; RIF: Rifampicin, SXT: Trimethoprim/sulfamethoxazole; TET: Tetracycline.
[a] = studies on more than one animal group.

Table S2. Molecular typing reports of *S. aureus* isolated from the NTO cavities of various free-living wild animals.

Reference	Animal Species (Description) (country)	Sample size	No. of *S. aureus*	No. of MRSA	No. *mecA*-MRSA	No. *mecC* MRSA	*spa* types of MRSA[a]	ST of MRSA (number)	CC of MRSA[a] (number)	*spa* types of MSSA	ST of MSSA[a] (number)	CC of MSSA (number)
**Wild Mammals (WM excluding NHP and rodents)**												
[5][a]	Iberian ibex, red deer, and wild boars (Spain)	1120	188	0	0	0	NT	NT	NT	**t034** (2), **t571** (1), t7304 (2), t002 (4), t1736 (3), t528 (16), t843 (2), t1535 (1), t098 (2), t127 (5), t607 (2), t1407 (2), t2601(1), t11223 (1), t6220 (1),	ST398 (3), ST130 (3), ST133 (2), ST5 (4)	NT
[18]	Wild Boars (Portugal)	45	15	1	1	0	**t899**	ST398	NT	t1533, t3750, t286, t14312, t14311, t10668, t3583, t3750, t11230, t10712, t11230, t10712	ST1 (2), ST3223 (4), ST3222, ST3220, ST2328 (2), ST133	NT
[19]	Red Deers (Spain)	65	16	11	0	11	t843 (4), t1535 (7)	ST1945 (11)	CC130 (11)	t2420 (5)	ST133 (5)	CC133 (5)
[21][a]	Wild mammals (Spain)	2506	NR	4	4	0	**t011** (2), **t1451** (1), t127 (1)	ST398 (3) ST1 (1)	CC398 (3) CC1 (1)	NT	NT	NT
[23]	Wil mammals (Spain)	103	23	4	1	3	t843 (3)	ST130 (3), ST1 (1)-*mecA*	CC130 (3), CC1 (1)	t1535 (5), t7174, t1125, t6386 (2), t6056 (3)	ST130 (6), ST5 (2), ST133 (3)	CC130 (6), CC5 (2), CC425 (2), CC133 (3)
[25]	Wild boar (Spain)	371	51	1	1	0	**t011** (1)	ST398	CC398	t3750 (13), t16741, t3583 (5), t742 (1), t11232 (3), t6292 (1), t11212 (2), t127 (3), t843 (2), t1094 (1), t12923 (1)	ST2328(13), ST2328 (2), ST133 (5), ST425 (7), ST1 (3), ST130 (1), ST3224, ST5 (5)	CC425 (7), CC1 (3), CC133(15), CC130 (2), CC5 (5)

[29]	Rabbit and hare (Spain)	363	71	34	0	34	NT	ST1945 (33), ST5823 (1)	CC130 (34)	NT	NT	NT
[30]	Wild Ungulate (Spain)	137	0	0	0	0	NT	NT	NT	NT	NT	NT
[31]	European brown hare, European otter, European hedgehog, Eurasian lynx (Germany)	40	5	5	0	5	t843 (2), t10513 (1), t3256 (1), t4335 (1)	ST130 (4), ST2620 (1)	NT	NT	NT	NT
[32]	Wild rabbits (Portugal)	77	4	0	0	0	NT	NT	NT	t272 (4)	ST121 (4)	CC121 (4)
[33]	Wild boar (Germany)	111	41	0	0	0	NT	NT	NT	t127 (15), **t571**, t742, t3369, t6902 t7674, t10885, t12042, t14149, t14999, t15001, t15002, t14149, t021, t1181, t091	ST1 (15), ST7, ST30, ST133, ST425 (10), ST804, ST890, ST3237, ST3238, ST3255, ST3369, ST804, ST7	NT
[34]	Wild Hedgehog (Sweden)	55	NR	35	0	35	t843 (17), t10751, t978 (3), t9111 (3), t15312 (4), t3391 (5),	NT	CC130 (20), CC2361(15)	NT	NT	NT

							t10893 (1), t11015 (1)					
[35]	Bats (Gabon and Cote d' Ivoire)	133	4	0	0	0	0	NT	NT	t15196, t15197 (3)	ST2984, ST3259, ST3301, ST3302	NT
[39]a	Shrews (China)	185	14	1	0	0	t15965	ST3323	NT	NT	NT	NT
**Non-human primates (NHP)**												
[43]	Free living chimpanzees (Zambia and Uganda)	62	36	0	0	0	NT	NT	NT	t1247 (16), t934 (9), t7723 (4)	ST2168 (16), ST15 (4), ST80 (9)	NT
[45]	Apes and Lemur (Madagascar)	19	7	0	0	0	NT	NT	NT	**t10694** (4), t127 (1), t189 (2)	ST1 (4), ST182, ST188 (2)	NT
[46]	Non-human Primates (Gabon, Cote d' Ivoire)	95	58	0	0	0	NT	NT	NT	t127, t6533	ST1, ST9, ST15, ST36 and ST152,	CC9, CC15, CC80, CC601
[47]	Primates (Nepal)	59	25	4	4	0	NT	ST22 (3), ST239 (1)	NT	NT	NT	NT
**Wild Birds (WB)**												
[5][a]	Eurasian griffon vulture (Spain)	40	2	0	0	0	NT	NT	NT	t7304 (2)	ST133 (2)	NT
[21][a]	Eurasian Griffon vulture (Spain)	40	NR	2	2	0	**t011** (2)	ST398 (2)	CC398 (2)	NT	NT	NT

[24]	Wild birds (USA)	114	10	3	2	0	t002, t008 (2)	ST5 (1), ST8 (2)	NT	t4735, t4368, t2603, t4634, t1059, t1166, t094	ST1959, ST2018	NT
[36]	Birds of prey (Buzzard) (Portugal)	16	1	0	0	0	NT	NT	NT	NT	ST30	CC30
[37]	Stork (Spain)	92	32	3	2	1	t843 (1), **t011** (2)	ST3061 (1), ST398 (2)	CC130 (1), CC398 (2)	**t571** (5), **t6606** (3), **t3625 (1)**	ST5 (12), ST398 (7), ST133 (5), ST2377 (1)	CC5 (12), CC398 (9), CC133 (5)
[38]	Cinereous vulture and Magpie (Spain)	324	15	13	1	12	t843 (11), t1535 (1), **t011** (1)	**ST1583 (3), ST398 (1), ST1945 (7), ST1581 (1), ST1571 (1)**	CC130 (12), CC398 (1)	t267 (1), t5998 (1)	ST97 (1), ST425 (1)	CC97 (1), CC425
**Wild Rodents (WR)**												
[12]	Free-Living wild rats (Germany and Czech Republic)	145	37	2	1	1	t843 (1), t685 (1)	NT	CC130 (1), CC30 (1)	t1048 (4), t208 (2), t14284 (12), t843 (6), t1535 (4), t3256 (6), t19038 (1)	NT	CC130 (20), CC49, (15)
[17]	Wild Rodents (Portugal)	204	38	6	3	3	t1535 (3), t747 (2) t018 (1)	ST1945 (3), ST22 (2), ST36 (1)	CC130 (3), CC22 (2), CC36 (1)	**t1451** (5), **t571** (1), t843 (7), t3256 (2), t516 (7)	ST398 (4), ST5926 (2), ST130 (7), ST1245 (2), ST1094 (7)	CC398 (6), CC130 (9)

[22]	Wild rodents and shrews (Germany, Czech and France)	295	45	1	0	1	t843 (1)	NT	CC130 (1)	t208 (11), t841 (1), t1736, t1773, t2311, t3058, t3830, t4189 (6), t9909, t15027	ST49 (17), ST88 (1), ST130 (1)	CC49 (17), CC88 (1), CC195, CC130 (1)
[39][a]	Rodents (China)	212	87	11	11	0	**t034** (1), **t011** (1), **t4552** (1), **t2582** (1), **t558** (1)	ST398 (4), ST1243 (2), ST59 (1), ST45 (1), ST1232 (1), ST1 (1), ST338 (1)	CC398 (5), CC59 (4), CC5 (1), CC45 (1)	NT	NT	NT

NB:

[a]In bold are indicated the *spa*-types associated to *S. aureus* isolates of the lineage CC398; in gray shadow are indicated the *spa*-types or CC types of *mec*C-MRSA isolates.

Key:

NT: not tested.

ST: Sequence Type.

CC: Clonal Complex

[a] = studies on more than one animal group.

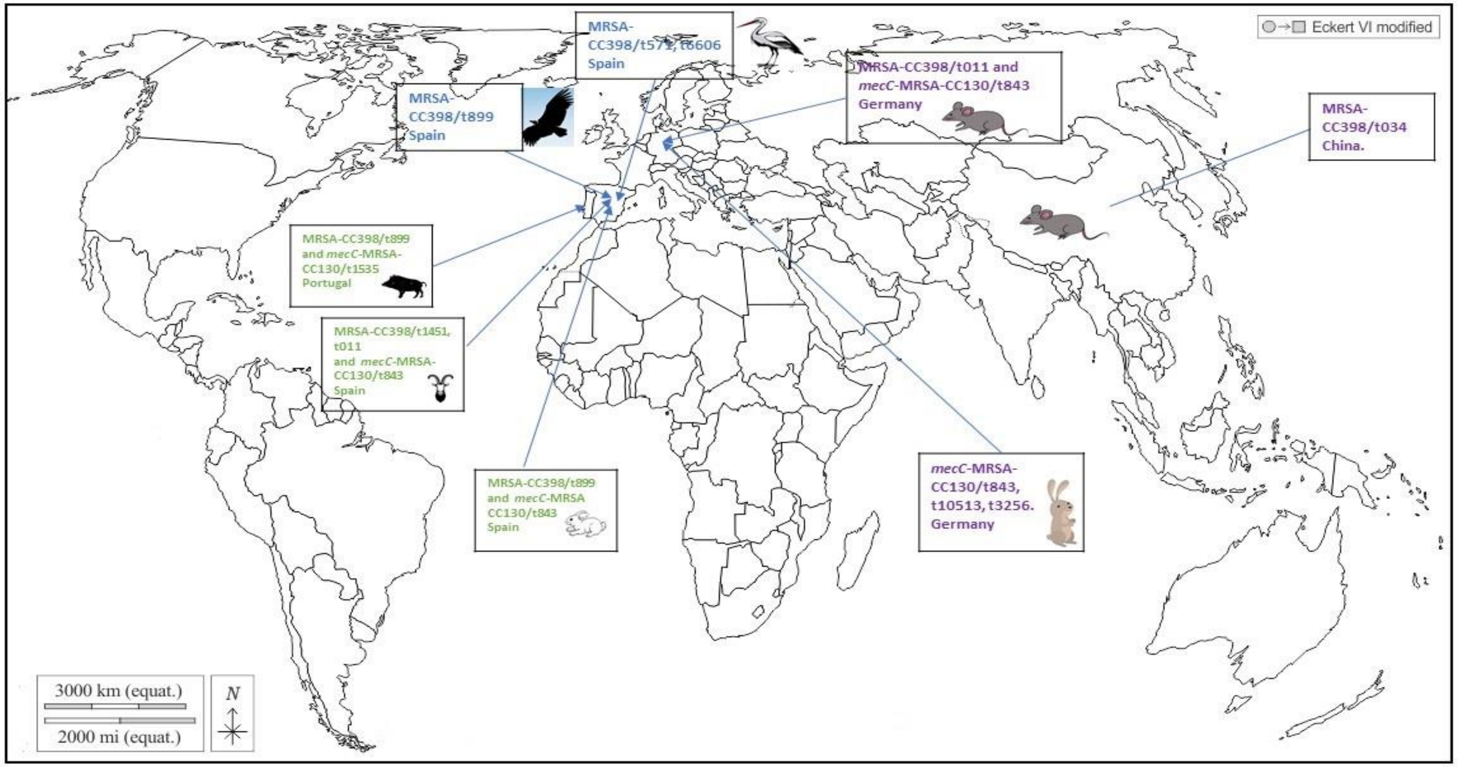

Figure S1. Geographic distribution of MRSA-CC398 and *mecC*-MRSA isolates detected in NTO cavities of wild animals (data obtained from tables 2 and 3).

Note: MRSA-CC398 and *mecC*-MRSA isolates were only reported in Europe and China (Raafat *et al* [12], Sousa *et al* [18], Porrero *et al* [5, 21], Ruiz-Ripa *et al* [23], Moreno-Grúa *et al* [29], Loncaric *et al* [31], Ruiz-Ripa *et al* [38], Ge *et al* [39]).
